# A hybrid PROSAIL inversion framework for winter wheat LCC using hyperspectral data and transfer learning

**DOI:** 10.3389/fpls.2026.1921769

**Published:** 2026-08-05

**Authors:** Xueqing Zhu, Juan Wang, Ying Nian, Jikai Liu, Xinwei Li

**Affiliations:** 1 College of Intelligent Manufacturing, Anhui Science and Technology University, Chuzhou, China; 2 College of Resources and Environment, Anhui Science and Technology University, Chuzhou, China; 3 Anhui Engineering Research Center of Smart Crop Planting and Processing Technology, Anhui Science and Technology University, Chuzhou, China; 4 Anhui Province Key Laboratory of Functional Agriculture and Functional Food, Anhui Science and Technology University, Chuzhou, China

**Keywords:** hyperspectral remote sensing, LCC, machine learning, PROSAIL, transfer learning, winter wheat

## Abstract

Leaf chlorophyll content (LCC) is a key indicator for assessing the photosynthetic capacity and nutritional status of winter wheat. Among traditional LCC estimation methods, empirical models lack a physical basis and have poor generalisability, while physical models are widely applicable but suffer from ill-posed inversion problems. Hybrid inversion methods, which integrate radiation transfer models such as PROSAIL with machine learning, offer both the interpretability of physical models and the efficiency of machine learning; however, they are still affected by the domain shift between simulated and measured data, which limits their generalisation performance. Transfer Component Analysis (TCA), a domain adaption method, can effectively alleviate this problem. In this study, hyperspectral and LCC data were collected in the field, and simulated data were generated using the PROSAIL model; a sensitivity analysis was conducted to identify LCC-sensitive bands. A genetic algorithm was applied to the measured data for band selection and, together with the results of the sensitivity analysis, yielded an optimal set of 30 characteristic bands for subsequent modelling. Three datasets were constructed: measured data only, a direct mixture of measured and simulated data, and a TCA-fused mixture of measured and simulated data. Four models—gradient boosting regression (GBR), random forest (RF), support vector regression (SVR) and deep neural network (DNN)—were developed for each dataset. The results show that: (1) the LCC-sensitive bands are concentrated in the 450–660 nm and 680–720 nm ranges; (2) the model built on the TCA-fused data (R² = 0.722, RMSE = 6.792) outperformed those built on the measured-only data (R² = 0.682, RMSE = 7.259) and the directly mixed data (R² = 0.616, RMSE = 7.976); (3) for the TCA-fused data, the four models differed considerably in accuracy, with SVR performing best (R² = 0.723, RMSE = 5.363), followed by RF (R² = 0.630, RMSE = 6.201) and GBR (R² = 0.575, RMSE = 6.650), whereas the DNN performed worst (R² = 0.388, RMSE = 7.947), probably owing to the limited sample size.

## 1 Introduction

Leaf chlorophyll content (LCC) is a critical photosynthetic pigment-related trait in winter wheat, and its content serves as an important indicator for evaluating assessing crop nutritional status and growth dynamics. Rapid and accurate estimation of winter wheat LCC is of great significance for guiding field management, achieving efficient agricultural production management, and promoting high-quality, high-yield winter wheat production (Wang et al., 2024d). Traditional LCC estimation primarily relies on destructive sampling and laboratory chemical analysis, which are time-consuming, costly, and unsuitable for large-scale, and continuous dynamic monitoring (Sonobe and Hirono, 2023). Remote sensing technology enables the rapid and timely acquisition of agricultural, environmental and meteorological information, and has been widely applied in the modern agricultural management (Wang et al., 2022), providing a viable method for rapid, non-destructive LCC estimation.

Currently, the main methods for remote sensing-based LCC technology include empirical modelling, physical modelling and hybrid modelling. The empirical modelling approach combines ground-based sampling data to establish an empirical regression relationship between raw spectral data or vegetation indices and LCC, thereby avoiding the need to explicitly simulate the complex process of photon transport within the canopy. Although empirical modelling can achieve high accuracy over small areas, it is susceptible to vegetation index saturation and the non-uniqueness of functional relationships (Liu et al., 2013), and factors such as remote sensing sensors, land cover types and coverage levels can all influence model results, resulting in a lack of portability and limited transferability (Fan et al., 2023).How to enhance the spatial generalisation ability of a model whilst reducing reliance on ground-based sampling has become the primary challenge facing empirical modelling methods.

The physical modelling approach simulates the radiative transfer process within the crop canopy based on rigorous physical processes and mathematical models. By simulating the crop canopy reflectance under different combinations of crop biophysical parameters, incident radiation and observation geometry, a model for LCC estimation is established (Wang et al., 2024d). Among these, the PROSAIL radiative transfer model comprehensively takes into account factors such as crop canopy structure, growth conditions, and the remote sensing observation environment; it is capable of accurately simulating crop canopy reflectance and is widely used in LCC estimation (Chen et al., 2025). Previous studies have utilised the PROSAIL model to estimate LCC for crops such as maize, rice, wheat, soybean, cotton, and potatoes, employing methods including vegetation indices, look-up tables, Bayesian networks and Gaussian process regression (Jacquemoud et al., 2009; Weiss et al., 2000). However, due to the inherent simplifications and assumptions of physical models, as well as uncertainties arising from sensor noise and data processing errors in observations, discrepancies exist between the model simulation results and field observations (Koetz et al., 2005). In particular, model simulations often yield similar or identical simulated reflectance values for combinations of multiple input parameters, leading to a ‘one-to-many’ ill-posed inversion problem when matching reflectance values to simulated parameters during inversion (Atzberger, 2004; Combal et al., 2003).This poses significant challenges for this method when applied to high-resolution, large-scale remote sensing data.

The hybrid modelling approach generates large simulation datasets based on radiative transfer models (RTMs), trains models using machine learning algorithms, and then applies these models to measured datasets, thereby reducing reliance on prior knowledge and the demand for ground-based measured data (Berger et al., 2018). The hybrid modelling approach combines the universality of physical models with the accuracy and efficiency of machine learning methods. Whilst maintaining computational efficiency, it adheres to the fundamental physical rules encoded in radiative transfer models (García-Haro et al., 2018) and is widely applied in crop parameter estimation (Zhao et al., 2025). Furthermore, hybrid models can not only adapt to complex data patterns but also preserve the interpretability of physical processes. They can effectively mitigate the issue of ill-posed inversion in physical models, whilst simultaneously addressing common problems in parameter inversion such as data redundancy, random noise, overfitting, and underfitting, thereby enhancing the stability and accuracy of inversion models (Zhou et al., 2024). In recent years, hybrid inversion models based on machine learning algorithms such as multi-layer perceptrons, support vector regression, random forests, and gradient boosting have gained widespread favour in crop parameter inversion (Verrelst et al., 2012). At the same time, these studies have demonstrated that hybrid models possess development potential and significant advantages in LCC inversion; however, they still face the problem of domain shift between simulated and measured data (Tuia et al., 2016). That is, there are differences in the distributional characteristics between the RTM simulation data and the measured data, which lead to a decline in the model’s generalisation performance on the measured data. .

Transfer learning is a cornerstone of modern machine learning and plays a crucial role in developing robust artificial intelligence systems suitable for open and dynamic environments (Zhou, 2022; Zhang et al., 2024b). Transfer learning enables artificial intelligence systems to reuse knowledge acquired from past tasks and generalise it to new tasks, thereby eliminating the need for extensive supervised data collection and model training from scratch, and significantly reducing time and resource costs. One of the primary challenges in the transfer process is the distributional disparity between the data of the new task and that of the source task (Sun et al., 2025). The transfer component analysis (TCA) method can effectively address this issue (Pan et al., 2011). By identifying a shared feature subspace between the source and target domains, the differences in distribution between the domains are minimised, thereby enabling effective knowledge transfer (Long et al., 2013). Existing research has shown that TCA has achieved good results in tasks such as remote sensing image classification, crop type identification, and soil property prediction (Verrelst et al., 2015), but its application in the field of crop LCC inversion remains limited. Therefore, how to incorporate transfer learning methods such as TCA into hybrid crop LCC inversion models to mitigate the domain shift between simulated and measured data has become an important research direction for improving the performance of hybrid models.

**Figure 1:**
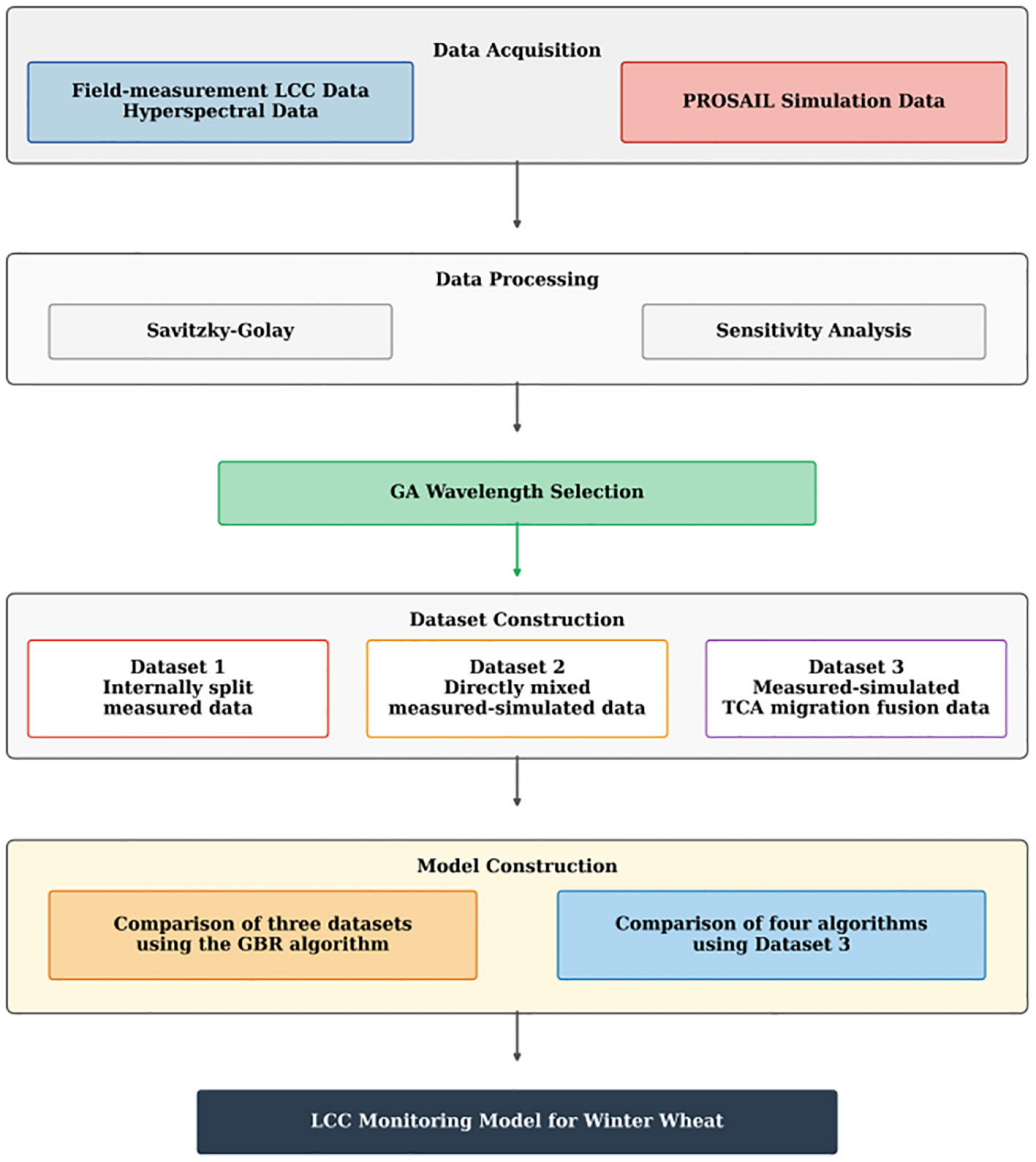
Research flowchart.

In light of this, this study utilises PROSAIL simulated data alongside field-measured hyperspectral and LCC data. It employs genetic algorithms for wavelength selection and applies the transfer component analysis method to construct LCC hybrid estimation models based on different datasets and algorithms (**Figure 1**), aims: (1) To generate a simulated dataset covering various combinations of crop parameters based on the PROSAIL model, and to use a genetic algorithm to screen for the optimal wavelength combinations thtat are sensitive to LCC, thereby reducing data dimensionality and improving model efficiency; (2) To conduct a comparative analysis of the performance differences of machine learning algorithms—including multi-layer perceptrons, support vector regression, random forests, and gradient boosting—in LCC inversion; (3) To introduce TCA, construct a transfer learning framework from the source domain (simulated data) to the target domain (measured data), and evaluate its effectiveness in mitigating domain shift issues; (4) To comprehensively compare the combined effects of different algorithms and transfer strategies, and select the optimal LCC hybrid estimation model, thereby providing valuable insights for the optimisation of LCC inversion techniques and crop growth monitoring practices.

## 2 Materials and methods

### 2.1 Study area

**Figure 2:**
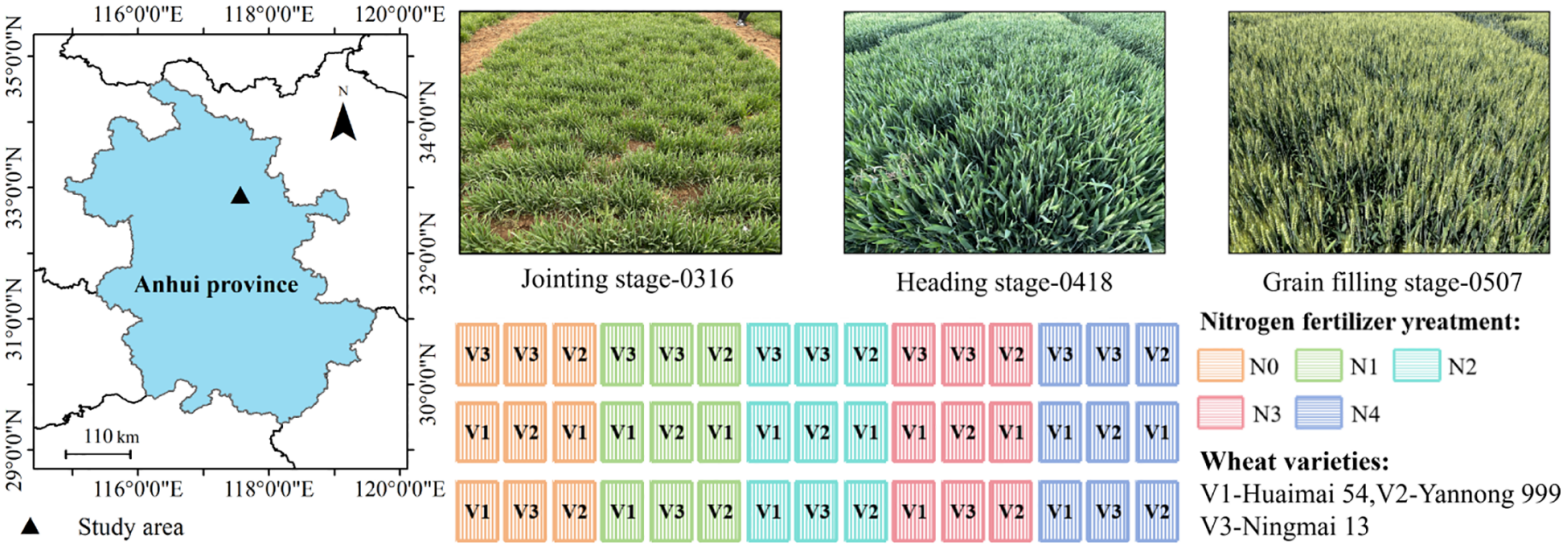
Location of the study area.The study area is located in Fengyang County, Anhui Province. It comprises 45 plots, three varieties and five nitrogen gradients, and data were collected at three growth stages.

The study area is situated in Fengyang County, Chuzhou City, Anhui Province (32°53′15″N, 117°33′52″E), as shown in the **Figure 2**. The study area has a subtropical monsoon climate, with an average annual temperature of 16.3 °C and an annual precipitation of 904.7 mm (Anhui Statistical Yearbook, https://tjj.ah.gov.cn/ssah/qwfbjd/tjnj/index.html). The study area was divided into 45 plots, comprising five nitrogen gradient treatments, three varieties, and three replicates. Each plot measured 6 m in length and 4.4 m in width, with a plant spacing of 30 cm; seeds were sown using row sowing. .

### 2.2 Collection of field data

#### 2.2.1 SPAD data collection

**Figure 3:**
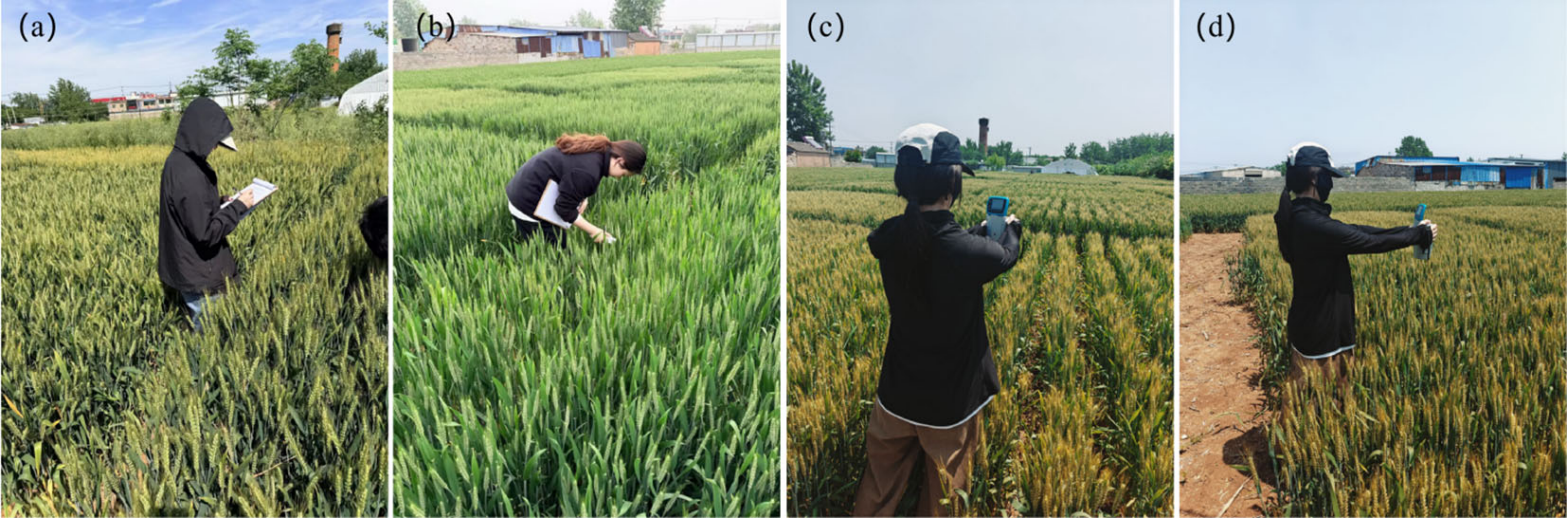
Data acquisition in the study area: **(A)** SPAD data recording; **(B)** field acquisition of SPAD data; **(C, D)** field acquisition of hyperspectral data.

Field sampling for this experiment was carried out on 16 March, 18 April, and 7 May 2024. The TYS-4N plant nutrition analyser (Zhejiang Topyun Nong Technology Co., Ltd., China) was used to obtain Soil and Plant Analysis Development (SPAD) data from canopy leaves at each growth stage (**Figure 3**). Within each plot, 10 winter wheat plants with uniform growth were selected. Measurements were taken at the midpoint of the second leaf of each plant, and the average avlue of the 10 samples was taken as the SPAD value for that plot. Based on the research findings of Cerovic et al. (2012), Equation 1 was used to convert the SPAD data into LCC data, with LCC expressed in μg/cm².

(1) LCC=(90*SPAD)/(144−SPAD)

#### 2.2.2 Acquisition of hyperspectral data

Hyperspectral data acquisition was carried out concurrently with LCC measurements. Field measurements were conducted in 45 plots within the study area using a FieldSpec^®^ HandHeld 2™ portable ground-based spectrometer (Analytical Spectral Devices, Boulder, Colorado, USA). The spectral measurement range was between 325 and 1075 nm, with a resolution of 1 nm. Measurements were carried out on clear, windless days between 10:00 and 13:00 local time. Three sample points were measured in each plot, yielding a total of nine spectral curves; the average of these were taken as the raw spectral data for that plot. During measurements, the spectrometer probe was always held perpendicular to the winter wheat canopy at a distance of 0.5 m. Calibration was performed using a standard white board before each data collection session and subsequently every three plots.

### 2.3 Acquisition of PROSAIL simulation data

The PROSAIL model is a radiative transfer model widely used in the field of vegetation remote sensing retrieval. It consists of two coupled sub-models: the PROSPECT model and the SAIL model (Li et al., 2025b). The coupled PROSAIL model accounts for radiative transfer processes at both the leaf and canopy scales simultaneously. By inputting various leaf parameters, it accurately simulates the radiative transfer process within the vegetation canopy (Wang et al., 2025). The ranges for Cab and LAI in the model were determined based on the actual conditions of the study area, whilst the ranges and default values for other parameters were selected in accordance with relevant literature pertaining to the crop types within the study area.

### 2.4 Sensitivity analysis

Sensitivity analysis is a method used to assess the extent to which model parameter inputs influence the model outputs. When performing inversion modelling using the PROSAIL radiative transfer model, different parameters contribute to the model outputs to varying degrees, which may affect modelling accuracy; therefore, conducting a sensitivity analysis on the input parameters is crucial (Lu et al., 2021). Sensitivity analysis is typically divided into local sensitivity analysis and global sensitivity analysis. Local sensitivity analysis examines the extent to which a change in a single parameter affects the model outputs whilst other parameters remain constant; this method is straightforward, rapid, and easy to implement, but it cannot account for interactions between parameters (Wang et al., 2025). Global sensitivity analysis, on the other hand, is better suited to examining the impact of parameters and their coupled effects on the model outputs (Cao et al., 2025).

The Extended Fourier Amplitude Sensitivity Test (EFAST) method employs model variance analysis, whereby the output variance of the model is generated by the individual input parameters and the interactions between them, thereby reflecting the degree of sensitivity of the input parameters. The EFAST method offers advantages such as high robustness, computational efficiency, and a low number of required samples (Wang et al., 2024c), and is capable not only of analysing the sensitivity of individual parameters but also of accounting for coupling effects between parameters; it is therefore suitable for analysing the parameter sensitivity of high-dimensional non-linear models (He and Yang, 2013).

The EFAST global sensitivity analysis was performed on the parameter values specified for the PROSAIL model using SimLab 2.2.1 software. Parameter settings were made according to **Table 1**; parameter names and ranges were entered individually into the SimLab software, with parameters set to follow a uniform distribution. The EFAST method was selected, with 5,000 iterations, ultimately generating 4,991 parameter combination datasets; A Python programme was written to input the generated parameter combination data into the corresponding PROSAIL model code; the model was then run to generate simulated data in a file format readable by SimLab software; within SimLab, the parameter combination data was coupled with the simulated data, and a sensitivity analysis was performed using the Monte Carlo method to obtain the sensitivity analysis results.

**Table 1:** PROSAIL model parameters.

Parameter type	Input parameter	Description	Range	Fixed
Leaf parameters	Cab(μg/cm²)	Chlorophyll a+b concentration	17-67	/
	N	Leaf structure index	1~2	/
	Cw(cm)	Equivalent water thickness	0.005-0.02	/
	Cm(g/cm²)	Dry matter content	0.001-0.01	/
	Cant	Anthocyanin content		0
	Cbrown	Brown pigment		0
Canopy parameters	LAI(m²/m²)	Leaf area index	0.8-6.3	/
	ALA/ALIA(°)	Average leaf inclination angle	40-70	/
	SZA/tts(°)	Solar zenith angle		30
	VZA/tto(°)	Observer zenith angle		0
	SAA(°)	Solar azimuth angle		0
	Psoil	Dry/Wet soil factor	0.01-0.1	/
	Rsoil	Soil brigthness factor		1
	Hspot	Hot spot parameter		0.1

### 2.5 Genetic algorithms

A genetic algorithm (GA) is an optimisation search algorithm that simulates the mechanisms of natural selection and heredity, and is used to solve complex optimisation problems (Yi et al., 2025). The fundamental principle behind genetic algorithms is natural selection in the process of biological evolution, whereby random selection and optimisation of the genome are achieved through recombination and mutation (Goldberg, 1989). As a global stochastic search algorithm, genetic algorithms are capable of searching the entire solution space for the optimal parameter solution to the cost function, subject to prior information constraints. This reduces the likelihood of the inversion results falling into local minima and ensures the rationality of the inversion results (Xu et al., 2019). Compared with general deterministic search algorithms (such as the simplex method and the conjugate direction set method), genetic algorithms exhibit superior global convergence performance (Song et al., 2009; Tang et al., 2002). Consequently, this study employs a genetic algorithm to minimise the cost function, thereby screening for the optimal band combination for LCC from high-dimensional raw spectra.

Within the GA, the performance of band subsets is evaluated using 5-fold cross-validation on the training set; sufficient training samples are required to ensure the stability and reliability of this procedure. Consequently, all data from 2024 were stratified by growth stage, with a training-to-test set ratio of 8:2, thereby retaining more samples for model training and band evaluation. At the same time, SVR was selected as the unified evaluation method. By varying only the band combinations, interference from algorithmic differences in the wavelength selection results was eliminated. Using RMSE as the cost function, and through iterative optimisation via selection, crossover, and mutation operations, the algorithm automatically screened 30 optimal band combinations from the full set of bands that were most sensitive to LCC, thereby enhancing the model’s accuracy and stability (Equations 2)

(2) $${\text{RMSE}}_{{\text{λ}}_{\text{i}}}=\sqrt{\frac{\sum_{\text{i}=1}^{\text{n}}{\left({\text{R}}_{\text{meas}}({\text{λ}}_{\text{i}})-{\text{R}}_{\text{sim}}({\text{λ}}_{\text{i}})\right)}^{2}}{\text{n}}}$$

Where RMSE ${\text{λ}}_{\text{i}}$ denotes the difference for band ${\text{λ}}_{\text{i}}$; Rmeas( ${\text{λ}}_{\text{i}}$) and Rsim( ${\text{λ}}_{\text{i}}$) represent the measured and simulated reflectance, respectively; and n is the total number of bands included in the calculation.

### 2.6 Model construction

TCA is a classic transfer learning method for domain adaptation (Pan et al., 2008), used to learn transferable feature components across different datasets, with the aim of preserving data attributes and reducing differences in data distributions across domains (Wan et al., 2022). This study employed linear TCA, with the n_components set to 18 and a regularisation coefficient (λ) of 10.0.

GBR is an ensemble learning method that constructs a strong predictive model by sequentially combining over multiple weak learners. Its core principle involves fitting the residuals of the previous model in each training round, which is equivalent to optimising along the negative gradient of the loss function, thereby continuously improving overall predictive accuracy. GBR gradually enhances model performance by iteratively training weaker learners and adjusting sample weights at each iteration, and performs exceptionally well in handling non-linear relationships and data noise (Wang et al., 2024a). The GBR hyperparameters are: learning rate [0.05, 0.1], n_estimators [100, 150], and max_depth [2, 3].

RF is an ensemble learning technique that improves accuracy and robustness by combining the predictions of multiple decision trees (Svetnik et al., 2003). Each decision tree is trained on a random subset of the data and features, and the final prediction is derived by averaging the predicted values from each tree. This method utilises the diversity among the decision trees to mitigate overfitting and enhance generalisation ability, making random forests a versatile and powerful tool for addressing various predictive modelling problems (Rodriguez-Galiano et al., 2015). In particular, for the RF model, the hyperparameters were set as n_estimators [100, 200] and max_depth [8, 12].

SVR is a kernel-based method that constructs a regression function by fitting a subset of the training data within a specified margin (Drucker et al., 1996). SVR extends the concept of support vector machines to regression tasks, with the objective of fitting a function within a specified tolerance range of the actual target values (Smola and Schölkopf, 2004). SVR aims to strike a balance between the model’s complexity and its ability to fit the data by minimising the deviation between the predicted values and the actual target values whilst maintaining the specified tolerance (Li et al., 2025a). The RBF (Radial Basis Function) kernel is selected, with a penalty coefficient C = [1, 10], gamma = [scale/auto], and a loss threshold ϵ = [0.1, 0.2].

Deep neural networks (DNN) consist of multiple layers, including an input layer, several hidden layers and, an output layer. When each layer of a DNN is fully connected, every neuron in one layer is connected to every neuron in the next layer. By systematically increasing the number of hidden layers, the model gains the ability to recognise and learn more complex patterns, thereby improving the predictive performance of regression or classification tasks under specific conditions (Liu et al., 2021). The DNN employs a three-layer network architecture, with two hidden layers following the input layer; the number of neurons in these layers is 128 and 64, respectively, using the ReLU activation function. The initial learning rate is 0.001, with the Adam optimiser set to 0.001, epoch = 100, and batch_size = 16.

All experiments were conducted in a Python environment, primarily utilising the following library versions: Python 3.12.4, NumPy 1.26.4, pandas 3.0.2, scikit-learn 1.8.0, and TensorFlow 2.19.1. The experiments were run on a Windows 11 operating system with an Intel Core i7-13700H processor and 32 GB memory.

### 2.7 Evaluation metrics

The model’s performance was evaluated using the coefficient of determination(R²) (Equation 3), root mean square error (RMSE) (Equation 4) and mean absolute error (MAE) (Equation 5). The closer R² is to 1, the higher the model’s predictive accuracy; the smaller the RMSE and MAE, the better the model’s performance. The calculation formulas are as follows:

(3) $${\text{R}}^{2}=1-\frac{\sum_{\text{i}=1}^{\text{n}}{\left(\text{y}-{\text{y}}_{\text{i}}\right)}^{2}}{{\left(\text{y}-\bar{\text{y}}\right)}^{2}}$$

(4) $$\text{RMSE}=\sqrt{\frac{\sum_{\text{i}=1}^{\text{n}}{\left(\text{y}-{\text{y}}_{\text{i}}\right)}^{2}}{\text{n}}}$$

(5) $$\text{MAE}=\frac{\sum_{\text{i}=1}^{\text{n}}|\text{y}-{\text{y}}_{\text{i}}|}{\text{n}}$$

Where: y and yi represent the observed chlorophyll content and the model-derived predicted value for a sample, respectively; $\bar{\text{y}}$ and $\bar{{\text{y}}_{\text{i}}}$ and represent the observed mean and the mean of the derived values for the samples, respectively; n is the number of samples.

## 3 Results

### 3.1 PROSAIL model simulation data

**Figure 4:**
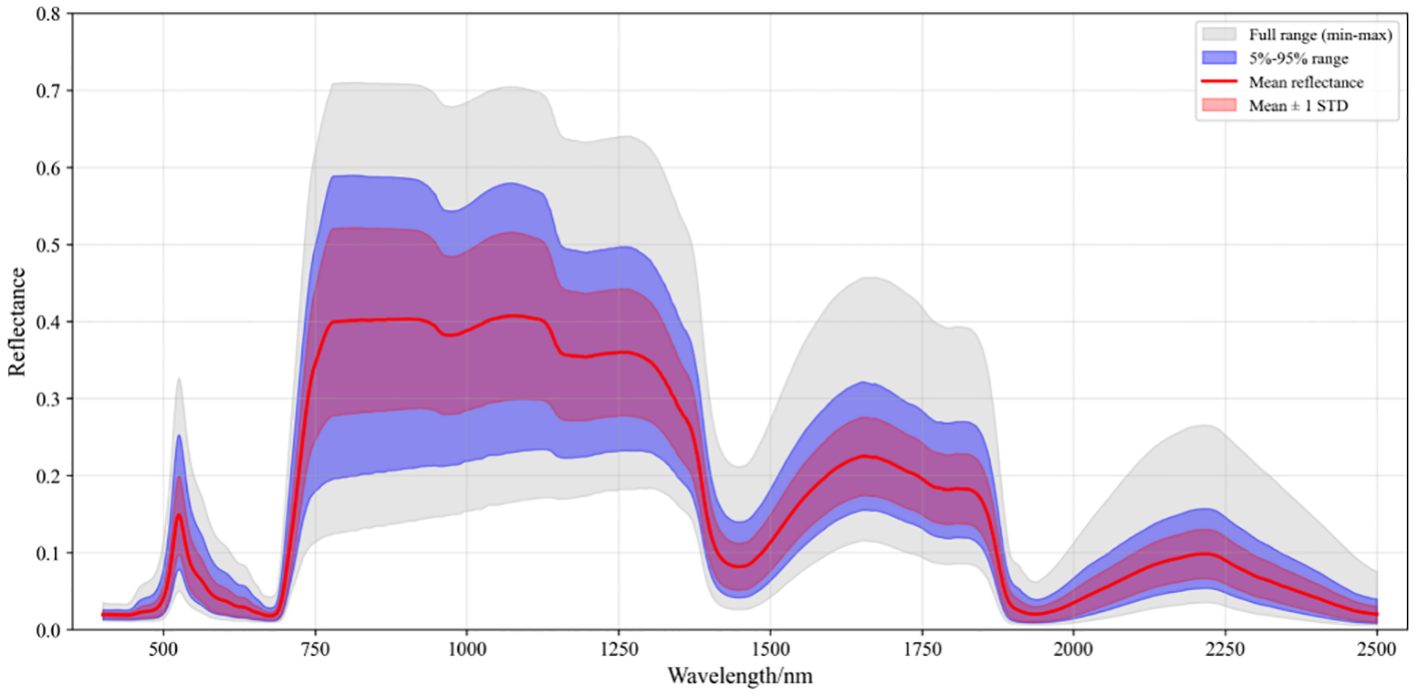
Canopy reflectance spectra simulated by the PROSAIL radiative transfer model under various biochemical and biophysical parameter combinations.

With the parameter distributions specified in **Table 1**, this study simulated canopy reflectance spectra covering the visible and infrared bands from 400 to 2500 nm, producing a total of 4,991 simulated spectral samples (**Figure 4**). Within the visible range, spectral characteristics are predominantly controlled by chlorophyll absorption near 500 nm and 670 nm, which result in a green reflectance peak at 550 nm and a red-edge transition at 700 nm. Decreases in chlorophyll concentration caused by leaf senescence will eliminate the reflectance valley near 670 nm and the steep red-edge transition at 700 nm on the spectra. For wavelengths longer than 900 nm, leaf water content dominates spectral variation, forming five typical spectral minima at 970 nm, 1180 nm, 1450 nm, 1930 nm and 2490 nm.

### 3.2 Sensitivity analysis

**Figure 5:**
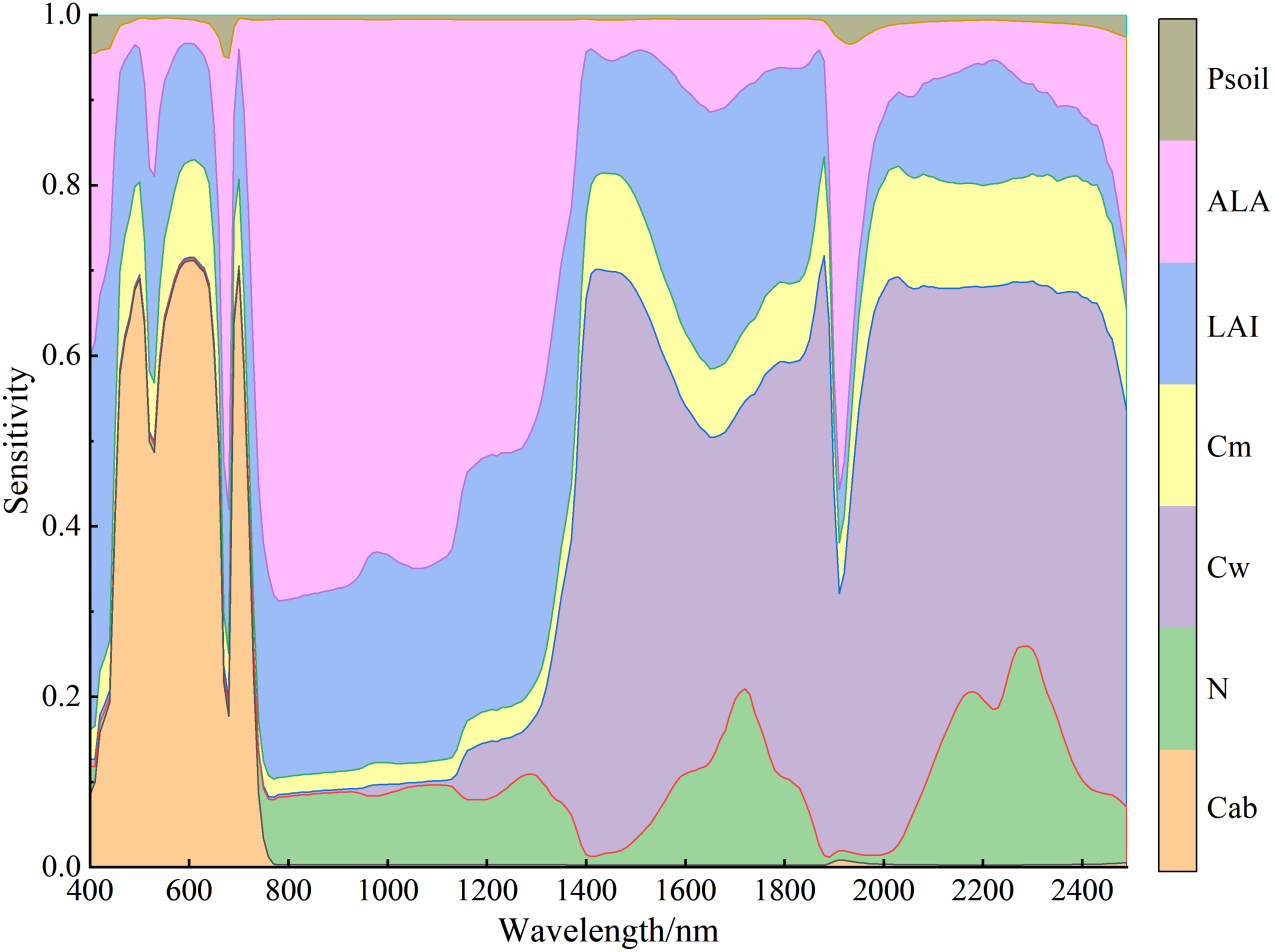
EFAST total-effect sensitivity analysis of PROSAIL model parameters.

Results of the total-effect sensitivity analyses (**Figure 5**) indicate that ALA and LAI contribute more substantially than Cab across 400–450 nm. Cab dominates spectral contributions in the 450–660 nm and 680–720 nm ranges, whereas its contribution decreases at 670–680 nm owing to the interference LAI. For wavelengths ranging from 780 to 1300 nm, ALA yields the greatest sensitivity, followed by LAI and N; Cab displays negligible sensitivity throughout this interval. In the 1300–2500 nm range, Cw becomes the most sensitive parameter.

### 3.3 Feature selection using genetic algorithms

**Figure 6:**
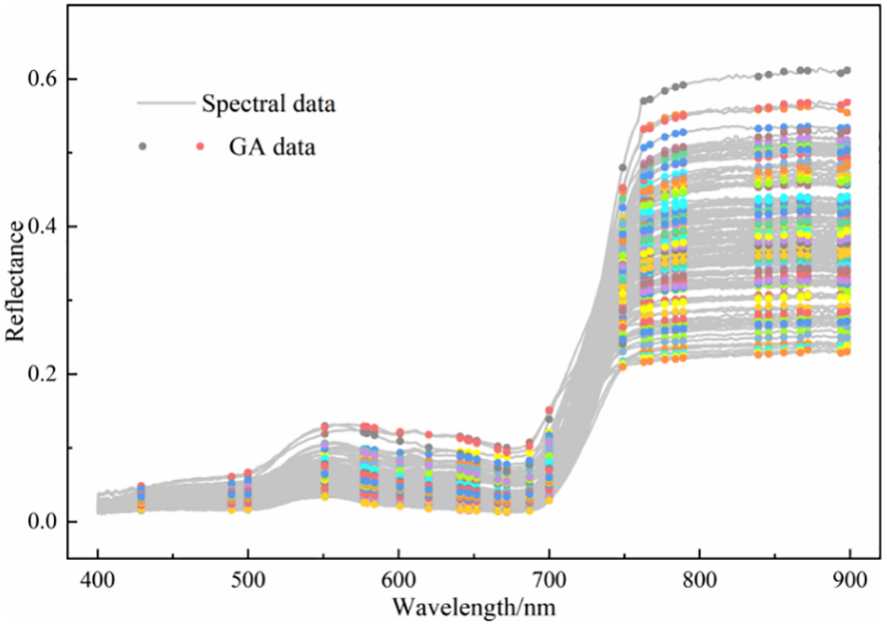
Convergence curve of the genetic algorithm for optimal hyperspectral band selection.

The measured spectral data within the 325–400 nm and 900–1075 nm bands suffer from severe atmospheric interference and a low signal-to-noise ratio; therefore, this study selected spectral reflectance data within the 400–900 nm range for subsequent analysis. Genetic algorithms were employed to select bands from the 400–900 nm spectral range, ultimately identifying 30 sensitive bands. The distribution of sensitive bands varies across different combinations. As shown in the results of Section 2.2, the LCC-sensitive bands are concentrated in the 400–780 nm range; therefore, the combination of bands with the highest concentration within this range was selected as the GA result (**Figure 6**): 429, 489, 500, 551, 577, 579, 584, 601, 620, 641, 646, 647, 652, 666, 672, 687, 700, 749, 763, 767, 777, 784, 789, 839, 846, 856, 867, 872, 894, 898.

### 3.4 Self-validation of PROSAIL model simulation data

**Figure 7:**
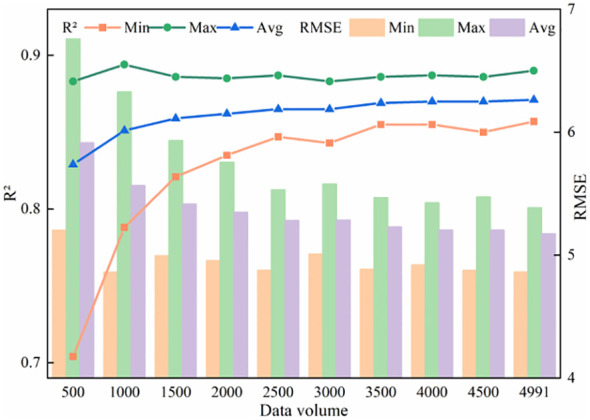
Self-validation of modelling results using different volumes of PROSAIL simulation data under the GBR algorithm.

Numerous studies have utilised the PROSAIL model to generate simulated spectral datasets for crop parameter estimation; the number of simulated data selected varies across studies, ranging from 3,000 to 100,000 data points. To validate the feasibility of the 4,991 simulated samples used in this study, a self-validation test was conducted using the GBR algorithm: Ten dataset sizes were defined: 500, 1,000, 1,500, 2,000, 2,500, 3,000, 3,500, 4,000, 4,500 and 4,991. Within each dataset size, the data were randomly split into training and test sets at a ratio of 3:1, and the process was repeated 100 times. The statistical results are shown in **Figure 7**. As the number of simulated samples used in modelling increased, the mean R² showed an upward trend, whilst the mean RMSE showed a downward trend. Both evaluation metrics reached their optimal values when the sample size reached 4,991, demonstrating the rationality of the simulated sample size used in this study.

To further demonstrate the reliability of the complete simulated dataset containing 4,991 records and the stability of the modelling, whilst maintaining the same internal data splitting method, and to avoid conclusions being affected by the uncertainty associated with a single algorithm, the RF method was used for repeated self-validation. The process was repeated 100 times, and results from 10 randomly selected runs are presented here. As shown in **Table 2**, under multiple random splits, the model’s R² was consistently above 0.90 and the RMSE was consistently below 4.6, indicating that the simulated data themselves possess high accuracy and that the model demonstrates good stability and reproducibility.

**Table 2:** Modelling results for the complete set of 4,991 simulated samples used for self-validation.

Test no.	R²	RMSE
1	0.905	4.465
2	0.901	4.568
3	0.905	4.466
4	0.913	4.332
5	0.905	4.407
6	0.909	4.343
7	0.902	4.517
8	0.904	4.499
9	0.904	4.474
10	0.913	4.205

### 3.5 Model development

**Figure 8:**
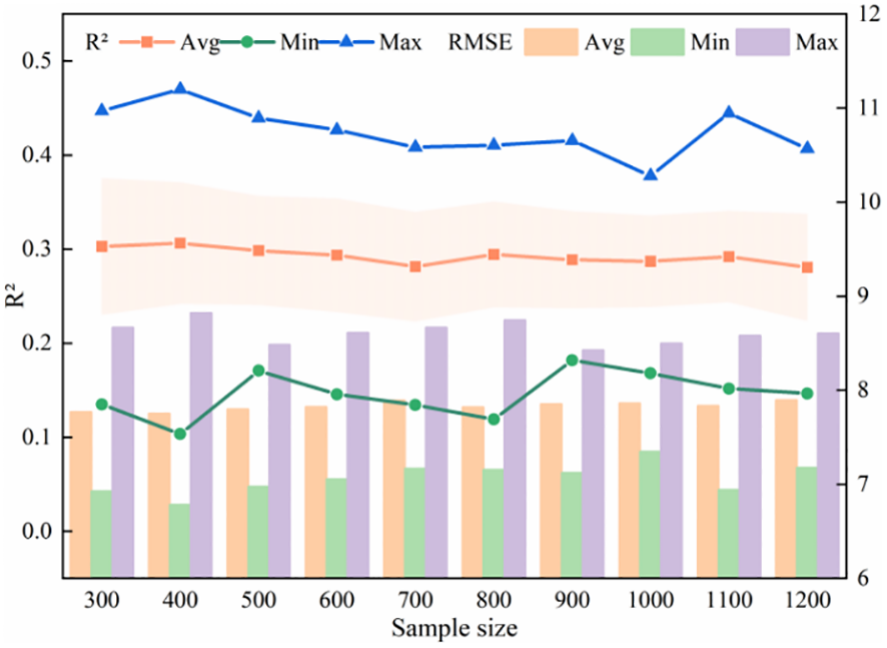
PROSAIL modelling results for the optimal number of samples. The orange pattern in the figure represents the standard deviation of R².

This study adopted three data combination strategies: internal partitioning of measured data, direct mixing of measured and simulated data, and transfer fusion of measured and simulated data. The above results indicate that, within a pure simulated-data self-validation framework, 4,991 records represented the optimal scale for simulated data. However, when applying the entire set of simulated data directly to the three different data combination strategies, multiple results from the four algorithms showed that the R² for Dataset 3 was negative, indicating significant domain distribution discrepancies between simulated and measured data; directly mixing all simulated samples would result in negative transfer. Consequently, experiments were conducted to identify the optimal sample size for cross-domain fusion of simulated and measured data, involving a comparative analysis of 10 groups with different sample sizes. Each set was repeated 100 times. The results are shown in **Figure 8**. The mean R² remained relatively stable within the range of 300–500 (0.298–0.306), whilst the standard deviation decreased monotonically as the sample size increased.

Based on this, three data combination strategies were defined for this study: (1) stratified sampling within the real-world dataset, with 95 samples used for the training set and 40 for the test set; (2) training with 400 simulated data points and 95 measured samples, and testing with 40 measured samples; (3) using the TCA transfer learning method, 400 simulated samples were treated as the source domain and 135 measured samples as the target domain for feature fusion; the resulting 400 fused simulated data points and 95 samples points formed the training set, whilst the remaining 40 real data points served as the test set.

#### 3.5.1 Comparison of different datasets

**Figure 9:**
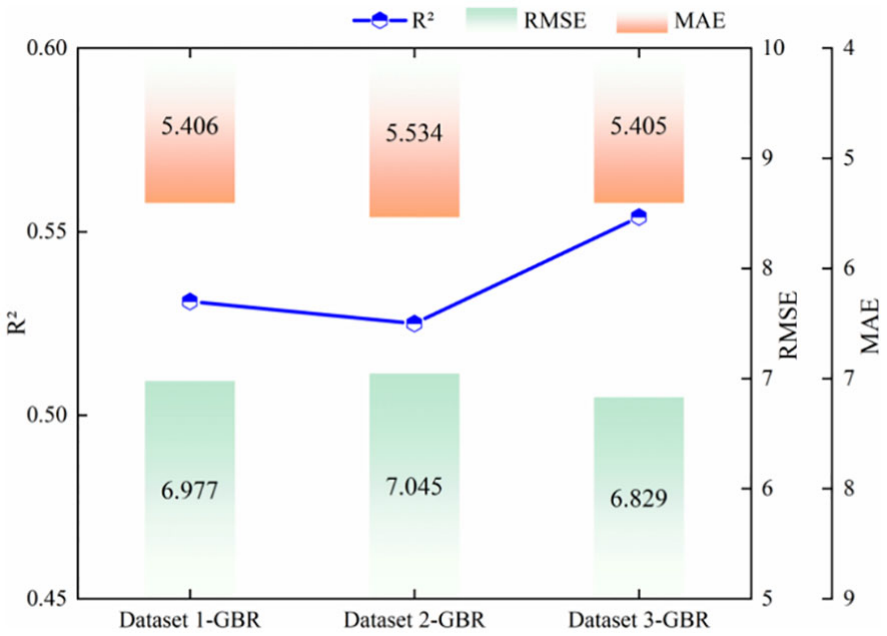
Comparison of modelling accuracy across different datasets. Dataset 1, Dataset 2 and Dataset 3 correspond to the internally partitioned measured dataset, the direct mixed measured–simulated dataset, and the transfer fusion dataset combining measured and simulated data, respectively.

**Figure 10:**
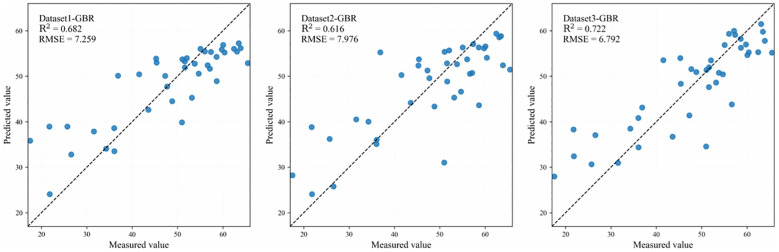
Measured and predicted LCC derived from the GBR algorithm based on different datasets.

To ensure consistent experimental conditions, the GBR model was used to construct the three data combination strategy models. As shown in **Figure 9** the modelling accuracy (R²) for the method based on internal partitioning of measured data was 0.532, with an RMSE of 6.9767 and an MAE of 5.4058; compared with the traditional model based solely on measured data, the model constructed using the direct mixture of simulated and measured data showed a 0.7% decrease in R², a 6.8% increase in RMSE and a 12.8% increase in MAE, with no improvement in predictive accuracy. The GBR model based on the TCA method not only exhibited lower error than the other models but also improveed predictive accuracy, achieving a 2.2% increase in R² compared with the best-performing method among the other two approaches, whilst reducing the RMSE by 14.8% and the MAE by 0.01%. To better illustrate the comparison between different datasets, a scatter plot of observed and predicted values was plotted using the results from a single run. **Figure 10** shows that the R² for the measured data is 0.682 and the RMSE is 7.259; for the data obtained by directly mixing simulated and measured data, the R² is 0.616 and the RMSE is 7.967; for the TCA-fused dataset, the R² is 0.722 - a 4% improvement over the measured data and a 10.6% improvement over the directly mixed data. with an RMSE of 6.792, representing a 46.7 percent reduction compared to the measured data and a 118.4 percent reduction compared to the directly combined data.

#### 3.5.2 Comparison of different algorithms

**Figure 11:**
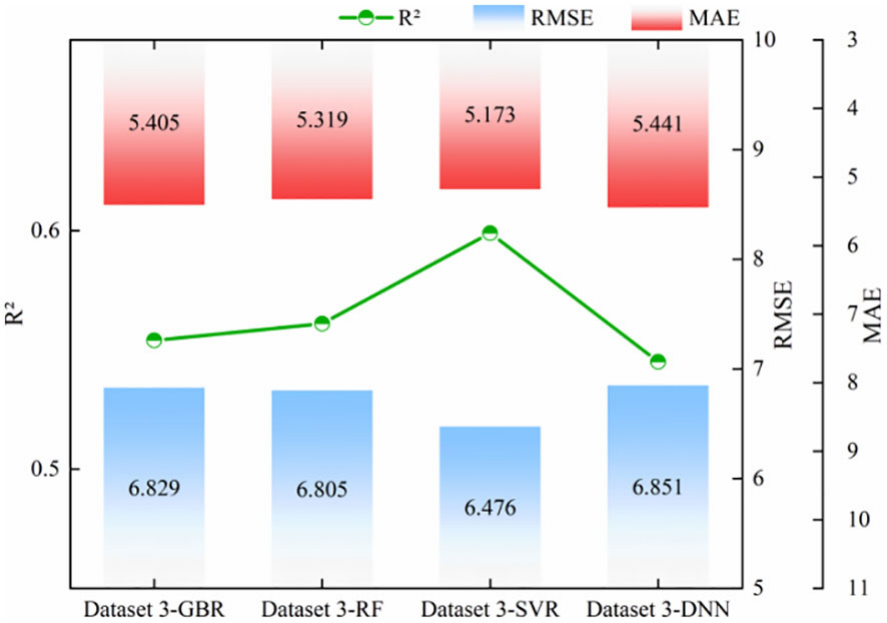
Mean modeling accuracy of different algorithms based on the TCA fusion dataset.

The above experiments demonstrate that the dataset processed via TCA fusion achieved satisfactory performance. To verify its adaptability and stability under different modelling algorithms, a multi-algorithm comparison was conducted using GBR, RF, SVR and DNN (**Figure 11**). Among these, SVR performed best, with an R² of 0.554, an RMSE of 6.829 and an MAE of 5.405; RF came second, with an R² of 0.561, an RMSE of 6.805 and an MAE of 5.319; GBR and DNN performed relatively less effectively, with R² values of 0.599 and 0.545 respectively, RMSE values of 6.476 and 6.851 respectively, and MAE values of 5.173 and 5.441 respectively.

To verify the statistical significance of performance differences among all models, nonparametric statistical tests were performed on the results of 100 independent replicates (S1). First, the Kruskal–Wallis test revealed significant differences among the algorithms in R² (H = 14.46, p=0.002), RMSE (H = 16.20, p=0.001), and MAE (H = 10.30, p=0.016). Subsequently, pairwise comparisons were conducted via the Wilcoxon signed-rank test, with the Bonferroni method adopted to correct for multiple comparisons (adjusted significance threshold α=0.0083). The pairwise test results indicated that SVR achieved significantly higher R² values than GBR (p<0.001, d=0.47), RF (p<0.001, d=0.50), and DNN (p<0.001, d=0.49). Meanwhile, SVR yielded significantly lower RMSE values than GBR (p<0.001, d=0.49), RF (p<0.001, d=0.53), and DNN (p<0.001, d=0.47), as well as significantly lower MAE values relative to GBR (p<0.001, d=0.37) and DNN (p<0.001, d=0.40). No statistically significant differences in any evaluation metric were detected among GBR, RF, and DNN.

**Figure 12:**
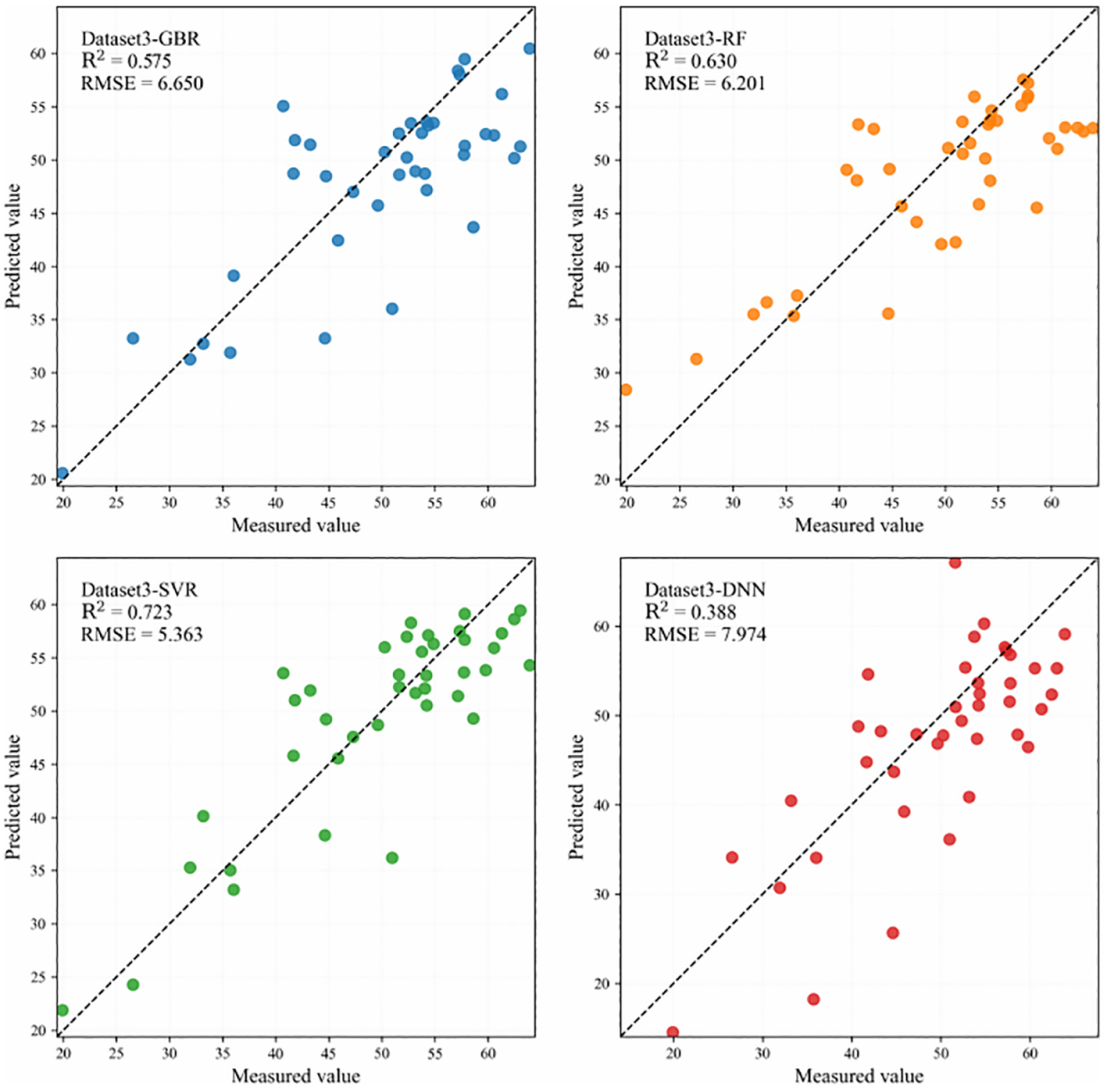
Measured and predicted LCC values for different algorithms based on the TCA-fused dataset.

**Figure 12** shows the scatter plots of observed and predicted LCC values for the four algorithms based on TCA-fused data, representing the results of a single run. Among these, SVR achieved an R² of 0.723, representing an improvement of 9.3% over RF (0.630), 14.8% over GBR (0.575) and 33.5% over DNN (0.388). The experiments demonstrate that, although the algorithms exhibited different levels of performance, they all achieved good inversion results on the dataset following TCA fusion, and all outperformed the internal partitioning scheme of the measured data. This fully validates the compatibility of the ‘measured–simulated transfer fusion’ strategy with different algorithms, indicating that this fusion method can systematically compensate for the insufficient scale of the measured data and enhance the model’s ability to estimate LCC.

## 4 Discussion

### 4.1 Accuracy of PROSAIL simulation data

**Figure 13:**
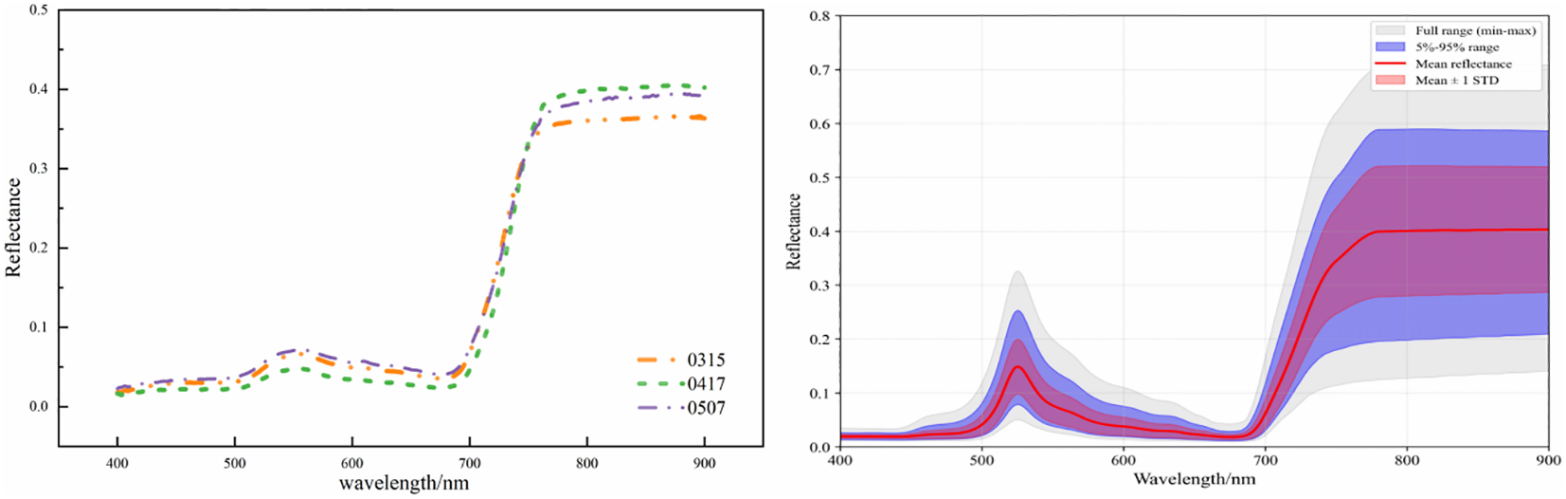
Comparison of Measured Spectra and PROSAIL-Simulated Spectra across Different Growth Stages.

The **Figure 13** presents spectral curves of PROSAIL-simulated and field-measured reflectance over the 400–900 nm range. The simulated and measured spectra share similar overall spectral patterns: a prominent reflectance peak occurs within 500–600 nm, a distinct absorption valley formsnear 680 nm due to strong chlorophyll absorption, reflectance rises steeply from 680 to 750 nm to produce the “red-edge” effect, and reflectance remains relatively stable from 750 to 900 nm.

Nevertheless, notable discrepancies exist between the two datasets. For field measurements, the green-band reflectance peak occurs at 550 nm; reflectance values across all growth stages range from 0 to 0.15, with an average below 0.1. In contrast, PROSAIL simulations reach the green reflectance peak at 520 nm, with reflectance spanning 0.05-0.35 and a mean value exceeding 0.1. This mismatch can be explained by differences in model parameters and the underlying physical processes.

**Figure 14:**
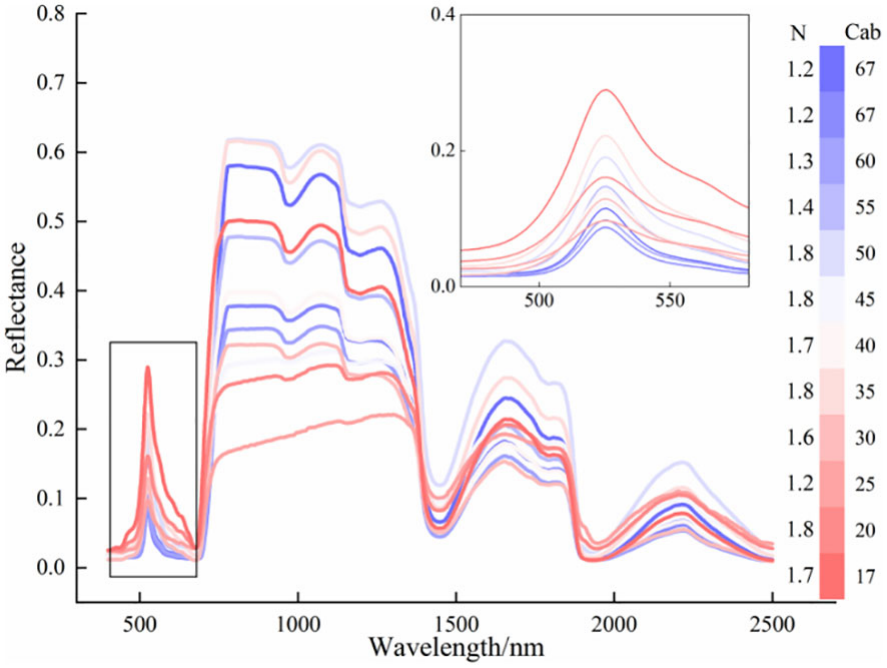
Simulated spectral curves at different levels of Cab and N.

There exists a coupling effect between Cab and N, and both parameters simultaneously influence reflectance spectra. As shown in **Figure 14** reflectance rises with decreasing Cab, and the green reflectance peak shifts toward shorter wavelengths (blue shift). Similarly, higher N values produce a response similar to that caused by reduced Cab: the green peak near 550 nm increases almost proportionally with increasing nitrogen concentration.

Consistent with the results reported by Jurise (Udal et al., 2020) et al., higher chlorophyll content reduces the magnitude the magnitude of the green reflectance peak and shifts the peak wavelength from 550 nm to 520 nm. Furthermore, Jurise et al. demonstrated that increasing anthocyanin and brown pigment concentrations can reduce the green reflectance peak. In the present study, both anthocyanin and brown pigment concentrations were set to 0 in the PROSAIL parameter configuration, which may contribute to the higher simulated green reflectance peak. The sensitivity analysis results (**Figure 5**) further support this interpretation. At 520 nm, the spectral response is affected by multiple parameters, with structural parameters exerting a stronger influence than biochemical parameters. LAI becomes the dominant factor, showing increased sensitivity, whereas the influence of Cab decreases, resulting in reduced sensitivity.

### 4.2 Analysis of wavelength selection results

**Figure 15:**
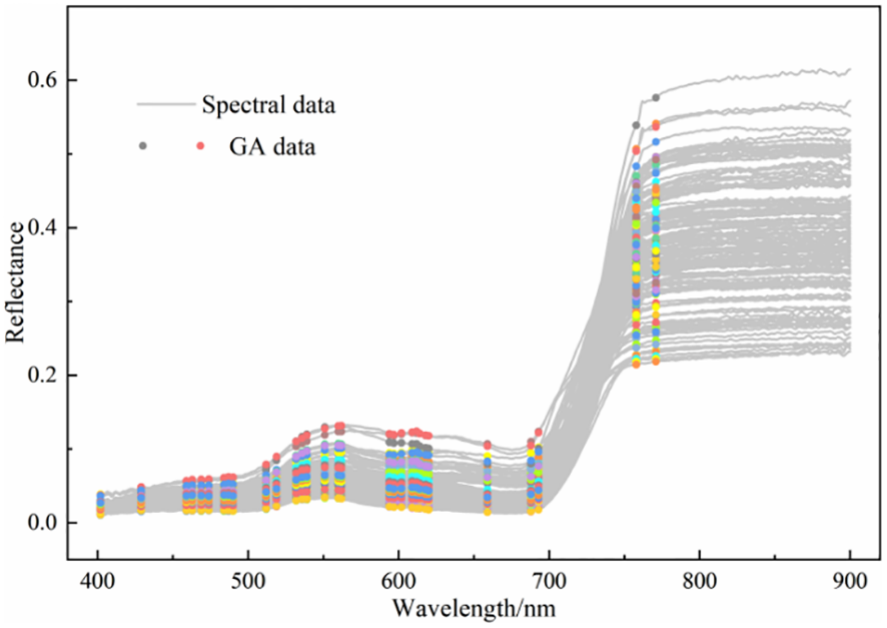
Spectral bands selected following weighting by the genetic algorithm.

**Figure 16:**
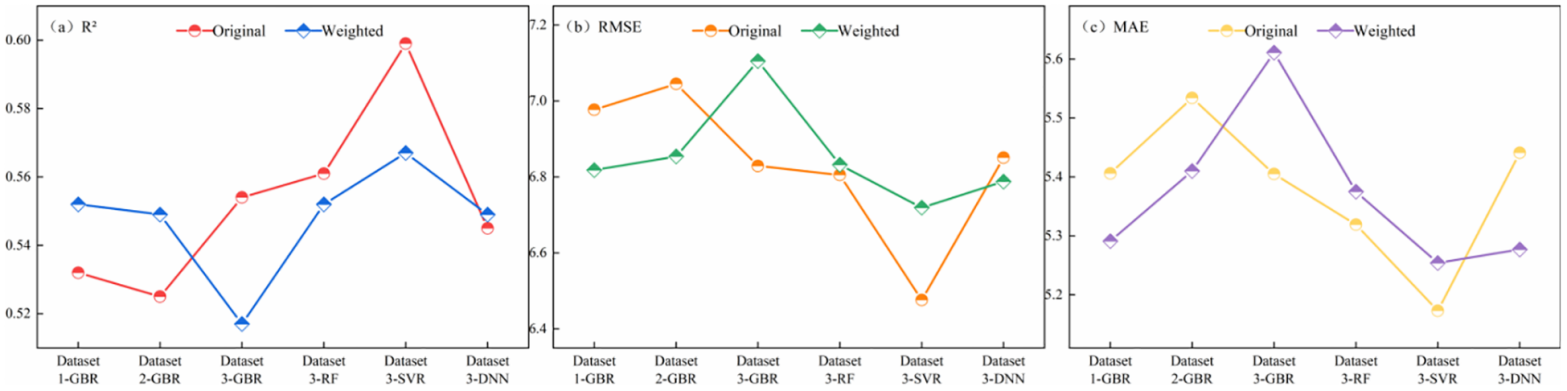
Comparison of results from the weighted genetic algorithm and the original genetic algorithm.

Genetic algorithm (GA)-based wavelength selection involves randomness. Results from multiple independent runs revealed substantial variations in band distribution: some iterations concentrated selected bands within the visible region of 400–700 nm, while others exhibited a relatively uniform distribution of selected bands across the 400–900 nm range. After repeated trials, the band subset whose selected wavelengths clustered in the high-contribution interval of 400–780 nm was adopted as the final output. Nevertheless, the proportion of low-contribution bands (780–900 nm) relative to the total selected bands approached one-third. To reduce the influence of selection uncertainty, the 400–900 nm band was divided into four levels based on the sensitivity analysis results, and the genetic algorithm was weighted accordingly: (1) 750–780 nm; (2) 400–450 nm, 660–690 nm and 720–750 nm; (3) 520–530 nm; (4): 450–520 nm, 530–660 nm and 690–720 nm. The weights increased progressively, and the final GA screening results were 402, 429, 459, 463, 469, 474, 484, 487, 490, 512, 519, 532, 536, 539, 551, 560, 562, 594, 597, 602, 609, 612, 614, 618, 620, 659, 688, 693, 758 and 771, as shown in **Figure 15**. The selected bands are all distributed within the range of bands with high sensitivity analysis contributions(**Figure 15**), However, bands longer than 780 nm accounted for asubstantially reduced fraction. The model was constructed using the results of the weighted GA, and the results are shown in **Figure 16**.

A comparison of the results from 100 repeated modelling runs for the two sets of feature bands under three data schemes and four regression models revealed (**Figure 16**) that the predictive performance of the models did not show a significant improvement following GA-weighted optimisation; fluctuations in model accuracy remained within the range of statistical error. This may be because low-contribution bands exhibit a weak individual response; however, when combined with sensitive bands in the visible and near-infrared regions, they can provide supplementary information on crop canopy structure and background soil, thereby alleviating the issue of multicollinearity among individual spectral regions. Although the exclusion of these bands through weighted screening aligns with sensitivity principles, it disrupts the synergistic complementarity of the original band combination, preventing any improvement in model accuracy. More than 70% of the unweighted bands were already concentrated in the high-sensitivity range; whilst the application of GA weighting increased the concentration of features in sensitive bands, it proved difficult to further improve accuracy. This is consistent with the findings of Zanella et al. (2022), who, using a GA-based wrapper to increase the population size from 50 to 200 individuals and the maximum number of generations from 35 to 150, also failed to improve accuracy. Research by Wang et al. (2024b). has demonstrated that the TCA method can eliminate biases caused by different instruments or external conditions, thereby enhancing the model’s predictive capability; this is also a contributing factor.

### 4.3 Analysis of model accuracy

The modelling results of the three datasets based on the GBR method showed that the model incorporating TCA achieves the highest accuracy, whereas the model based on the direct mixing of simulated and measured data exhibited the lowest performance. This may be because the measured data are complex and contain non-linear features, and as the distributions of the simulated and measured data differ, direct mixing would introduce noise and distributional bias (Kita et al., 2023) Domain adaptation methods reduce distributional discrepancies between source and target domains by projecting their features into a common latent space, thereby improving feature transferability (Pan and Yang, 2010). The core of TCAis to employ Maximum Mean Discrepancy (MMD) (Borgwardt et al., 2006) as a criterion for minimising marginal distributions differences between source and target domains (Zhou et al., 2023), thereby alleviating domain shift between simulated and measured datasets.

A comparison of model accuracies using the TCA method revealed that SVR performed best, followed by RF, whilst GBR performed relatively poorly, consistent with the findings of Chang et al. (2023). This may be related to the performance of the models themselves.

SVR is a non-linear algorithm. It maps features into a high-dimensional space via the RBF kernel function (Seko et al., 2017) and employs regularization parameters (C, ϵ) to alleviate overfitting. SVR presents strong adaptability to small-sample, high-dimensional nonlinear datasets. Since its decision boundary is determined by only a small set of support vectors, it can effectively eliminate disturbances caused by redundant information in high-dimensional spectral data (Nematov and Hojamberdiev, 2025).

RF is an ensemble learning method that generates a final prediction by averaging the predictions of multiple decision trees. It exhibits strong robustness against outliers. However, the stepwise prediction surface of tree-based models may introduce extra variance in regions with continuous variations within the spectral feature space. GBR is an advanced version of the boosting method and can be applied to various differentiable loss functions (Friedman, 2001). However, due to its sequential iterative training nature, GBR may be more sensitive to noise accumulation and has a higher risk of overfitting under small-sample conditions; consequently, it performs less well than SVR and RF. Furthermore, as both GBR and RF employ bootstrap resampling to train decision trees, their generalisation ability and predictive performance may be poor when applied to small sample datasets (Lee et al., 2018).

The accuracy of the DNN model was lower than that of the three machine learning models. This is primarily attributable to the following reasons: on the one hand, the DNN network employed in this study comprised two hidden layers and had a relatively large number of trainable parameters. Parameter estimation and feature learning typically rely on a sufficient number of training samples. With a limited sample size, the model was unable to fit stable global feature relationships; during the training phase, it was susceptible to interference from random sample noise and local data features, resulting in reduced generalisation performance. On the other hand, the DNN did not incorporate strategies such as dropout or weight regularisation to suppress overfitting; training hyperparameters were set to fixed values, and no cross-validation or hyperparameter tuning was performed. Consequently, the model did not converge to an optimal solution, further limiting its predictive performance on small datasets.

### 4.4 Prospects and outlook

This study utilised hyperspectral measurements and PROSAIL-simulated spectra to construct multiple datasets and integrated TCA with several machine learning algorithms to develop an LCC estimation model for the 2024 growing season. During PROSAIL simulations, only selected key parameters were considered, while parameters such as anthocyanin content were assigned default values, resulting in discrepancies between the simulated and measured data. Future parameter configurations should better represent actual conditions; for parameters unavailable from field measurements, their values should be determined based on previous studies, crops characteristics and environmental conditions.

As demonstrated above, applying Transfer Component Analysis (TCA) to spectral data improved both model generalisation ability and predictive accuracy to different extents; however,a certain gap remains between the model performance and practical application requirements. Therefore, to further improve the accuracy and applicability of generalised models, improved TCA-based approaches could be explored for future model development (Wang et al., 2024b).

To address the issue of low accuracy in DNN, nested cross-validation and Bayesian hyperparameter optimisation should be employed in future work to conduct a global search for parameters such as learning rate, layer width and regularisation coefficient. This will enhance the DNN’s fitting capability and robustness in scenarios with small sample sizes, thereby fully exploiting the application potential of deep learning models.Okolie (2023) proposed a hybrid deep learning approach that substantially boosts model accuracy, where hyperparameter tuning is essential for performance improvement.

In this study, experiments were only conducted using single-year datasets. The overall sample size is limited, and the temporal sampling density is insufficient. The absence of multi-climate, multi-year and multi-region data restricts the model’s spatiotemporal generalization capacity. To address the above limitations, future work will acquire data covering different years, sites, crop varieties and sensors to build a multi-source dataset with spatiotemporal gradients. We will systematically evaluate the model’s predictive performance under diverse spatial and temporal conditions and further improve its practical application value. Zhang et al. (2024a) proposed LeafTNet, a deep transfer learning network. By integrating field measurements collected over two consecutive years, the network substantially improved the estimation accuracy of vertical LCC distribution. This finding demonstrates the critical role of dataset scale and temporal extension in boosting model generalization performance.

## 5 Conclusions

Based on PROSAIL simulation data, this paper utilised sensitivity analysis and genetic algorithms to identify wavelength bands sensitive to LCC inversion, constructed three datasets, and performed LCC inversion using four algorithms: GBR, RF, SVR and DNN. The main conclusions are as follows: Sensitivity analysis of the PROSAIL simulation data revealed that LCC exhibited the highest sensitivity in the 450–660 nm and 680–720 nm bands; however, in the 670–680 nm band, LCC sensitivity was reduced due to the influence of LAI; Modelling of the three datasets using the GBR model revealed that the accuracy of the TCA-fused data was superior to that of models based solely on measured data or direct blending models; Modelling of the TCA-fused dataset using the four algorithms showed that the SVR model achieved the highest accuracy, followed by the RF and GBR models, whilst the DNN model exhibited the lowest accuracy.

## Data Availability

The raw data supporting the conclusions of this article will be made available by the authors, without undue reservation.
